# Cardiovascular Biomarkers for Prediction of in-hospital and 1-Year Post-discharge Mortality in Patients With COVID-19 Pneumonia

**DOI:** 10.3389/fmed.2022.906665

**Published:** 2022-06-28

**Authors:** Lukas J. Motloch, Peter Jirak, Diana Gareeva, Paruir Davtyan, Ruslan Gumerov, Irina Lakman, Aleksandr Tataurov, Rustem Zulkarneev, Ildar Kabirov, Benzhi Cai, Bairas Valeev, Valentin Pavlov, Kristen Kopp, Uta C. Hoppe, Michael Lichtenauer, Lukas Fiedler, Rudin Pistulli, Naufal Zagidullin

**Affiliations:** ^1^University Clinic for Internal Medicine II, Paracelsus Medical University, Salzburg, Austria; ^2^Cardiovascular Disease in COVID-19, International Research Network, Ufa, Russia; ^3^Department of Internal Diseases, Bashkir State Medical University, Ufa, Russia; ^4^Department of Biomedical Engineering, Ufa State Aviation Technical University, Ufa, Russia; ^5^Scientific Laboratory for the Socio-Economic Region Problems Investigation, Bashkir State University, Ufa, Russia; ^6^Department of Urology, Bashkir State Medical University, Ufa, Russia; ^7^The Key Laboratory of Cardiovascular Medicine Research, Ministry of Education, Department of Pharmacy at the Second Affiliated Hospital, and Department of Pharmacology at College of Pharmacy, Harbin Medical University, Harbin, China; ^8^Department of Internal Medicine, Cardiology, Nephrology and Intensive Care Medicine, Hospital Wiener Neustadt, Wiener Neustadt, Austria; ^9^Department of Cardiology I, Coronary and Peripheral Vascular Disease, Heart Failure, University Hospital Munster, Munster, Germany

**Keywords:** COVID-19, long COVID-19, post-discharge mortality, cardiovascular biomarkers, sST2, VCAM-1

## Abstract

**Aims:**

While COVID-19 affects the cardiovascular system, the potential clinical impact of cardiovascular biomarkers on predicting outcomes in COVID-19 patients is still unknown. Therefore, to investigate this issue we analyzed the prognostic potential of cardiac biomarkers on in-hospital and long-term post-discharge mortality of patients with COVID-19 pneumonia.

**Methods:**

Serum soluble ST2, VCAM-1, and hs-TnI were evaluated upon admission in 280 consecutive patients hospitalized with COVID-19-associated pneumonia in a single, tertiary care center. Patient clinical and laboratory characteristics and the concentration of biomarkers were correlated with in-hospital [Hospital stay: 11 days (10; 14)] and post-discharge all-cause mortality at 1 year follow-up [FU: 354 days (342; 361)].

**Results:**

11 patients died while hospitalized for COVID-19 (3.9%), and 11 patients died during the 1-year post-discharge follow-up period (*n* = 11, 4.1%). Using multivariate analysis, VCAM-1 was shown to predict mortality during the hospital period (HR 1.081, CI 95% 1.035;1.129, *p* = 0.017), but not ST2 or hs-TnI. In contrast, during one-year FU post hospital discharge, ST2 (HR 1.006, 95% CI 1.002;1.009, *p* < 0.001) and hs-TnI (HR 1.362, 95% CI 1.050;1.766, *p* = 0.024) predicted mortality, although not VCAM-1.

**Conclusion:**

In patients hospitalized with Covid-19 pneumonia, elevated levels of VCAM-1 at admission were associated with in-hospital mortality, while ST2 and hs-TnI might predict post-discharge mortality in long term follow-up.

## Introduction

The novel coronavirus disease COVID-19 still represents a major clinical challenge to date. COVID-19 primarily involves the respiratory system and severe COVID-19 typically leads to bilateral pneumonia with consequent acute respiratory distress syndrome and high mortality rates (1). As the Spike protein S of the ACE-2 receptor serves as the binding site for SARS-CoV-2, cells with a high expression of the ACE-2 receptor are primarily affected by COVID-19, resulting in a broad range of clinical symptoms in COVID-19. Several clinical parameters including laboratory, electrocardiographic and radiology findings have been used to stratify mortality risk (1–3), yet monitoring parameters and long-term prognostic markers for COVID-19 remain scarce.

COVID-19 was reported to have a considerable impact on the cardiovascular system, including not only cardiac injury but also thromboembolic events. Given the correlation of myocardial injury and disease severity in COVID-19, novel cardiovascular biomarkers might prove to be an effective prognostic tool in COVID-19 patients.

High-sensitive troponin is released in response to myocardial injury and represents the gold standard for cardiovascular risk assessment (4). Previous studies studies found that myocardial injury, defined by increased serum cardiac high-sensitive Troponin (TnI) levels, was associated with a mortality rate of >50% in COVID-19 patients (5), while other studies have reported that 19.7% of all COVID-19 patients presented with myocardial injury. Moreover, these patients had a significantly higher mortality rate compared to COVID-19 patients with normal TnI (51.2 vs. 4.5%) (6). This finding is further emphasized by a meta-analysis which reported significantly higher TnI levels in severe COVID-19 compared with patients with mild COVID-19 (7).

Soluble suppression of tumorgenity-2 (sST2) is a member of the interleukin-1 receptor family and has recently emerged as a potentially useful tool for improving the assessment of cardiovascular disease (8–10). There are two isoforms of ST2, the membrane-bound ST2L, mediating cardio-protective effects through binding of its only known ligand IL-33 (11), as well as sST2, the soluble form of ST2, acting as a decoy receptor for IL-33, thereby inhibiting its potential cardio-protective effects (11). Of note, the IL-33/ST2 axis was also reported to play a potential role in the COVID-19 pathogenesis (12–14). sST2 itself was shown to be elevated in numerous clinical scenarios, including heart failure and cardiac remodeling, myocardial infarction, atherosclerosis as well as in inflammatory disease such as sepsis (8). Recent studies reported high levels of sST2 in COVID-19 patients, also correlating with CRP levels, a standard marker of COVID-19 activity (15). Similarly, Sanches et al. (16) reported a significant correlation between levels of sST2 and ICU admission as well as death, emphasizing its prognostic potential.

Several studies have revealed a potential link between COVID-19 and endothelial dysfunction. On this regard, also the term “Acute Vascular Distress Syndrome” was introduced, to account for the vascular pathophysiology in COVID-19 pneumonia (17, 18). This comprises vasoplegia along with a reduced ventilation-to-perfusion ratio, leading to an increased pulmonary blood flow with intrapulmonary right to left shunt (18). Accordingly, also an analysis of vascular biomarkers might contribute to diagnosis and prognosis in COVID-19.

Vascular cells adhesion molecule-1 (VCAM-1) is a protein acting as a cell adhesion molecule of vascular endothelium for lymphocytes, monocytes, eosinophils, and basophils. In a study on the effect of SARS-CoV-2 spike S1 protein on the activation of human lung microvascular endothelial cells, the incubation of HLMVEC with the S1 protein significantly induced the expression of VCAM-1 (19). In further trials, VCAM-1 was associated with COVID-19-related mortality (20). Moreover, in a meta-analysis (*n* = 2,213), VCAM-1 levels were linked with increased disease severity in COVID-19 patients (21).

Given the involvement of sST2 and VCAM-1 in inflammatory processes and the previous reports on their role in COVID-19, their potential impact on prognosis also in the long term seems plausible. Accordingly, we undertook a comparative analysis of novel cardiac biomarkers sST2 and VCAM-1 as well as high-sensitive Troponin I (TnI) in a 1-year follow-up of COVID-19 patients. We hypothesized that these cardiovascular biomarkers might be helpful in predicting 1-year mortality. Therefore, the aim of our work was to establish an possible association between the investigated cardiovascular biomarkers and in-hospital as well as post-discharge mortality, which could help to identify patients at risk.

Therefore, the aim of our work was to establish an association between biomarkers and mortality.

## Methods

The study was approved by the Local Ethical Committee (N5, 2020) and was performed in accordance with standards of good clinical practice and the principles of the Declaration of Helsinki. Written informed consent was obtained from all study participants prior to inclusion.

This prospective, non-randomized, single-center study enrolled 288 consecutive patients between June 2020 and September 2020, who were hospitalized due to COVID-19-associated pneumonia in a tertiary care center during the 1st “wave” of the COVID-19 pandemic. Initial diagnosis was established via CT-scan, PCR testing and specific antibodies at admission and COVID-19 specific medical treatment was applied according to current national COVID-19 guidelines (22).

Included were patients 18 years or older with confirmed COVID-19 disease and related pneumonia. Exclusion criteria included: acute ST-elevation myocardial infarction at admission, acute stroke at admission, active malignant disease within the last 3 years, and acute kidney failure at admission defined as glomerular filtration rate (GFR) <30 mL/min/1.73 m^2^, as well as pregnancy or lactation. In total, eight patients met the exclusion criteria and were therefore excluded (acute ST-elevation myocardial infarction: *n* = 2, acute stroke: *n* = 1, active malignant disease: *n* = 2, and acute kidney failure: *n* = 3).

**Figure 1:**
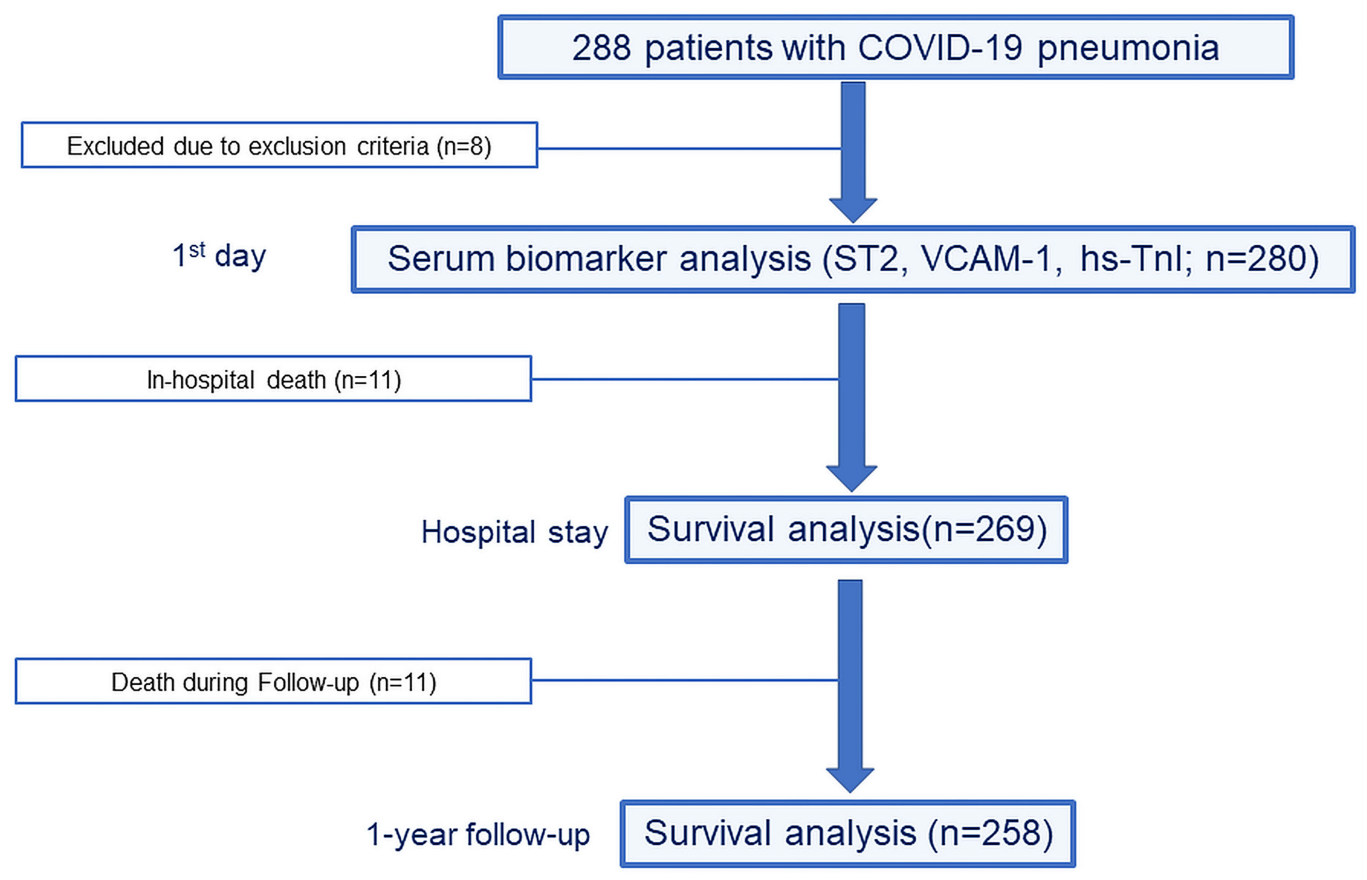
Design of the study.

Patient enrollment and the design of the study are presented in Figure 1. Upon hospital admission, venous blood was drawn, subsequently centrifuged, and the serum frozen at −20° C for further analyses. The concentration of biomarkers ST2, VCAM-1 and TnI was analyzed by enzyme immunoassay as indicated by the manufacturer (Thermo Fisher Scientific, USA for VCAM-1, Critical diagnostics, USA, for ST2, and Hema Ltd, Russia for TnI). In addition to the investigated biomarkers, further laboratory parameters were routinely measured according to current guidelines (Table 1).

**Table 1:** Characteristics of the study cohort.

**Parameter**	**Median (Q1; Q3) or %**
*N*	280
Gender, m/f	42.5 / 57.5%
Age, years	60 (50; 67)
Hospital stay + FU analysis, days	366 (357; 373)
BMI, kg/m^2^	27.9 (25.3; 32.6)
***Clinical presentation at admission***	
SpO_2_, % Body temperature at admission, °C SAP, mm Hg DAP, mm Hg HR, beats / min BR, breaths /min Lung tissue damage on CT, %	97 (95; 98) 36.7 (36.5; 37.45) 130 (120; 143) 83 (79; 90) 90.5 (76; 102) 19 (18; 20) 36 (22.5; 52)
***Relevant concomittant disease:***	
AH, % (*n*) DM, % (*n*) CKD, % (*n*) CHD, % (*n*) CHF, % (*n*) History of Stroke, % (*n*) Obstructive lung disease, % (*n*) History of AF, % (*n*)	38.9 (109) 7.5 (21) 1.4 (4) 6.4 (18) 2.1 (6) 0 (0) 7.5 (21) 0 (0)
***Laboratory parameters***	
Hb, dg/l WBC, *10^9^/l Platelets, *10^9^/l ESR, mm/sec CRP, mmol/l Procalcitonin, ng/ml Albumin, g/l CK, mmol/l Urea, mmol/l Creatinine, mmol/l GFR, ml/min/m^2^ D-Dimer, ng/ml Sodium, mmol/l Potassium, mmol/l	12.9 (119; 137.75) 4.55 (3.64; 6.65) 266 (172.25; 277) 29 (18; 40.75) 41.8 (18.8; 76.9) 0.09 (0.05, 0.16) 40.3 (37.8; 42.5) 120.0 (72; 213) 5.33 (4.38; 6.62) 85.8 (77.5; 99.1) 65.9 (57.1; 78.1) 641 (505; 824) 143 (141; 145) 4.2 (3.9; 4.4)
***Serum cardiovascular biomarkers***	
TnI, ng/mL VCAM-1, ng/mL ST2, ng/mL	0.03 (0.01; 0.07) 13.84 (9.79; 17.5) 52.5 (32.4; 77.9)
***Events during hospitalization***	
Oxygen therapy, % (*n*) NIV, % (*n*) ET, % (*n*) In hospital mortality, % (*n*) Total 1-year mortality, % (*n*) Hospital stay, days	64.3 (180) 6.4 (18) 3.2 (9) 3.9 (11) 7.9 (22) 11 (10; 14)

*AH, arterial hypertension; BA, bronchial asthma; CK, creatine kinase; CHD, coronary heart disease; CHF-congestive heart failure; CKD, chronic kidney disease; CRP- C-reactive protein; CT computer tomography; DBP, diastolic blood pressure; DM, Diabetes Mellitus type 2; ET-endotracheal intubation; ESR, erythrocytes sedimentation rate; Hb, hemoglobin, HR, heart rate;, MI, myocardial infarction; NIV-non-invasive mechanical ventilation; SBP, systolic blood pressure; CK, creatine kinase; ST2 - suppression of tumorigenicity 2; TnI – highly sensitive Troponin I, VCAM-1; vascular cells adhesion molecule-1; WBC, white blood count*.

A detailed medical history was obtained at admission for all enrolled patients, including current symptoms, as well as previous illnesses and current medications. The study was carried out between June 2020 and September 2020. Follow-up (FU) analysis was conducted during the acute phase of disease defined as the total hospital stay and after hospital discharge for a median of 366 days (356: 373) for the study endpoint with the help of the regional medical information analytical system “ProMed” (23). This web-based medical records system enables remote online monitoring of hospitalization discharge notes including death certificates. The study endpoint was defined as all-cause mortality as indicated by discharge notes and/or death certificate during the FU period. The FU endpoints were analyzed in September 2021.

**Figure 2:**
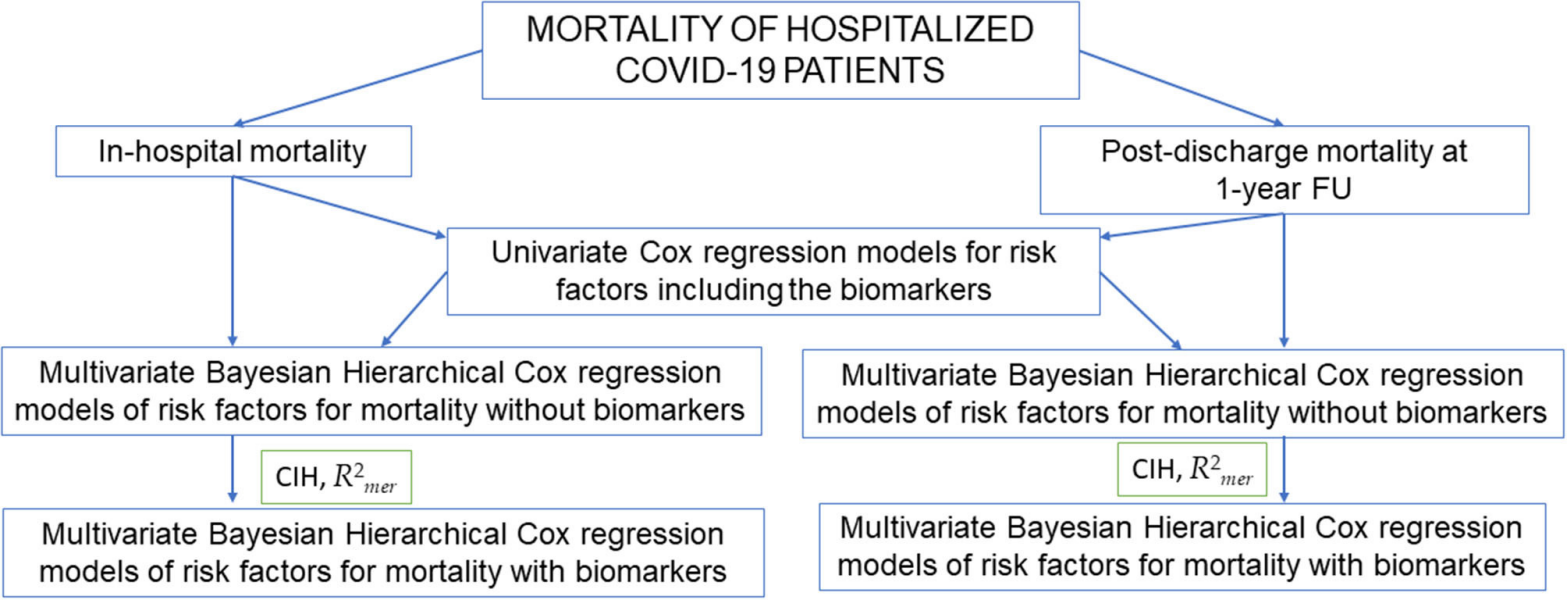
Mathematical model of the statistical analyses. ST2, soluble suppression of tumorigenicity 2; VCAM-1, vascular cells adhesion molecule-1; TnI, high sensitive troponin I.

The mathematical model for statistical analyses is summarized in Figure 2. All data were tested for normality. Normally distributed continuous variables were expressed as mean values (M) and standard deviations (SD). Non-normally distributed data were expressed as median (interquartile range Q1–Q3), and the non-parametric Mann-Whitney test was used for comparison between the two groups. Categorical variables were expressed as frequencies and proportions. Differences among groups were assessed with the Chi-squared test.

Prediction of biomarkers for prognosis in COVID-19 patients was analyzed as follows. At the preliminary stage of the analysis, univariate Cox proportional hazards models were evaluated. Biomarkers values were taken as independent variables. To remove the bias of estimates, age of the patients was also considered as a control factor. Then statistically significant factors with *p* > 0.1 were selected. The first multivariate model of survival was built for all-cause mortality during in-hospital stay, and the second for all-cause mortality after hospital discharge during a FU of 1 year. Gsslasso Cox Bayesian hierarchical models (24) were used to obtain reliable estimates of the models' coefficients. Application of this model was possible since it offers satisfactory results in the case of a correlation between cardiovascular risk predictors. The predictive power of the model was reassured by proximity to the Harrell concordance CI-index. *R*^2^_*mer*_ measure of explained risk was used as model quality metric. The interpretation of the model's results was based on the hazard ratio (HR) of survival for each risk predictor. To determine the predictive value of addressed serum cardiovascular biomarker as mortality risk predictors, two variants of models were considered: 1. a model which included all significant clinical and laboratory risk factors compared to 2. a model which additionally included the investigated cardiovascular serum biomarkers as risk predictors. The predictive value of both models was compared by quality metrics (Harrell concordance CI-index, measure of explained risk) of models with the inclusion/exclusion of biomarker values. A *p*-value < 0.05 was regarded as statistically significant. All statistical analyses were performed on R software (version 3.6.3, R Foundation for Statistical Computing, Vienna, Austria, https://www.r-project.org).

## Results

Demographic characteristics, clinical presentation at admission, relevant concomitant disease, and laboratory markers of our 280 hospitalized COVID-19 patients are presented in Table 1. COVID-19 relevant in-hospital therapies and relevant post-discharge therapies are presented in Supplementary Tables 1, 2. Arterial hypertension (AH) was the most frequent comorbidity. While 64.3% of patients required oxygen therapy, 6.4% needed support by non-invasive mechanical ventilation and 3.2% by endotracheal intubation. Hospital stays averaged 11 (10, 14) days, with an in-hospital mortality rate of 3.9%, whereas overall mortality at 1 year was 7.9%.

### In Hospital Survival Analysis

The in-hospital mortality rate was 3.9% (*n* = 11). This patient group was significantly older than patients surviving COVID-19 pneumonia (*p* < 0.001) and had lower oxygen saturation (*p* < 0.003) resulting in higher rates of non-invasive mechanical and invasive mechanical ventilation (both *p* < 0.001). Similarly, comorbidities were more common in these patients, including significant impairment of renal function. Among the investigated biomarkers, only VCAM-1 (*p* < 0.001) was significantly elevated in non-survivors, while no significant differences were observed for sST2 but a strong trend toward a statistical significance was evident for TnI (*p* = 0.05, Table 2).

**Table 2:** Comparison of COVID-19 in-hospital deceased vs. in hospital survivors.

**Parameter**	**Hospital survivals, (Q1; Q3) or %**	**Hospital deceased, (Q1; Q3) or %**	***p***
N, %	269 (96.1%)	11 (3.9%)	
Gender, m/f	114/155 (42.4%/57.6%)	5/6 (45%/55%)	0.820
Age, years	**59 (50**; **66)**	**71 (69.5**; **75)**	**<0.001**
BMI, kg/m^2^	27.83 (25.3; 32.0)	32.05 (27.1; 32.5)	0.235
***Clinical presentation at admission***			
SpO_2_, % Temperature at admission, °C SAP, mm Hg DAP, mm Hg HR, beats / min BR, breaths /min Lung tissue damage on CT, %	**97 (95**; **98)** 36.7 (36.5; 37.5) 130 (120; 140) 83 (79; 90) 91 (76, 102) 19 (18, 20) 36 (22; 52)	**95 (93.25**; **96.75)** 36,65 (36.6; 36.85) 144 (137.75; 148.75) 81 (80; 88) 83.5 (80, 104.25) 19 (18; 20) 38 (27, 43)	**0.003** 0.695 0.143 0.523 0.691 0.573 0.884
***Relevant concomitant disease***			
AH, % (*n*) DM, % (*n*) CKD, % (*n*) CHD, % (*n*) CHF, % (*n*) History of MI, % (*n*) History of Stroke, % (*n*) Obstructive lung disease, % (*n*) AF, % (*n*), % (*n*)	38.7 (104) 7.4 (20) **1.11 (3)** **5.2 (14)** 1.9 (5) 0 0 **7.8 (21)** 0	45 (5) 9 (1) **9 (1)** **36.4 (4)** 9 (1) 0 0 **0** 0	0.447 0.877 **0.027** ** <0.001** 0.057 - - **0.013** -
***Laboratory parameters***			
Hb, dg/l WBC, *10^9^/l Platelets, *10^9^/l ESR, mm/sec CRP, mmol/l Procalcitonin, ng/ml Albumin, g/l CK, mmol/l Urea, mmol/l Creatinine, mmol/l GFR, ml/min/m^2^ D-Dimer, ng/ml Sodium, mmol/l Potassium, mmol/l	129 (119; 127) **4.5 (3.6; 6.6)** **226.5 (173; 277.3)** 29 (18; 41) 41.6 (18; 77.9) **0.09 (0.05; 0.16)** 40.3 (37.8; 42.5) 124 (72; 213) **5.33 (4.38; 6.4)** **85.6 (76.9; 98.5)** **66.34 (57.5; 78.3)** 705.1 (505; 824) 143 (141; 145) 4.2 (3.9; 4.4)	13.8 (129.5; 144.75) **6.3 (5.6; 9.8)** **169 (129.5; 144.75)** 30.5 (19; 36.75) 26.5 (23.1; 51) **0.15 (0.11; 0.26)** 40.4 (37.8; 42.6) 99 (82.25; 155.5) **8.57 (8.5; 8.8)** **104.5 (97.3; 116.65)** **48.2 (44.2; 50.9)** 490 (460; 557) 142 (140.25; 143) 4.14 (3.93; 4.7)	0.217 **0.035** **0.011** 0.394 0.433 **0.005** 0.529 0.752 ** <0.001** **0.002** ** <0.001** 0.082 **0.022** 0.926
***Serum cardiovascular biomarkers***			
ST2, ng/mL VCAM-1, pg/mL TnI, ng/mL	52.26 (31.6; 77.64) **13.75 (9.57; 16.98)** 0.03 (0.01; 0.03)	72.35 (45.4; 72.4) **24.12 (17.7; 33.2)** 0.01 (0; 0.105)	0.762 ** <0.001** 0.050
***Events during hospitalization***			
Oxygen therapy, % (*n*) NIV, % (*n*) ET, % (*n*) Hospital stay, days	62.8% (169) 4.8 (13) 0.7 (2) 11 (10; 13)	100% (11) 45% (5) 55% (6) 13 (12; 19)	**0.012** ** <0.001** ** <0.001** 0.828

*AH, arterial hypertension; BA, bronchial asthma; CK, creatine kinase; CHD, coronary heart disease; CHF, congestive heart failure; CKD, chronic kidney disease; CRP, C-reactive protein; CT, computer tomography; DBP, diastolic blood pressure; DM, Diabetes Mellitus; ET, endotracheal intubation; ESR, erythrocytes sedimentation rate; Hb, hemoglobin; HR, heart rate; MI, myocardial infarction; NIV-non-invasive mechanical ventilation; SBP, systolic blood pressure; CK, creatine kinase; ST2 - suppression of tumorigenicity 2; TnI, highly sensitive Troponin I; VCAM-1, vascular cells adhesion molecule-1; WBC, white blood count. The p < 0.05 is marked bold*.

In the next step, investigated biomarkers were analyzed with the help of univariate Cox regression with age as the control variable. The endpoint in-hospital mortality using each biomarker was further analyzed with the help of univariate Cox regression, where age was the control variable. Table 3 presents coefficients of univariate Cox regression proportional hazards for investigated biomarkers according to in-hospital mortality. Of note, the most accurate mortality risk predictor was VCAM-1 (HR 1.086, *p* < 0.001). We further analyzed the differences in hospital survival rates in groups, according to the presence / absence of indicator or based on the normal / out of range laboratory and clinical parameters in patients by univariate Cox regression model analysis (Supplementary Table 3). The following variables were associated with hospital mortality with *p* < 0.1: age, SpO_2_, arterial hypertension, coronary heart disease, chronic heart failure, procalcitonin and GFR. A significance has been also shown for creatinine, urea, and existence of chronic kidney disease, but considering their direct association with GFR, only the latter was included into the multi-marker model. Furthermore, since WBC and platelets showed significant differences between non-survivors and survivors when applying the Mann-Whitney test (Table 2), we also decided to include them into the multivariable model, although no association (*p* > 0.1) was revealed when using the univariate Cox regression for patients with high WBC and low platelets (Supplementary Table 3). The variables WBC and platelets were added to the multivariable model as dummy variables (as 1 if WBC was > 8^*^10^9^ and also as 1 if platelets were <150^*^10^9^ or as 0 in the rest of the cases).

**Table 3:** Univariate Cox regression for biomarkers, associated with COVID-19 hospital mortality.

**Biomarker**	**Coefficient ±SE**	**Hazard ratio**	**CIH**	**CI**	***P***
ST2	−0.0003 ± 0.003	0.999	0.86	0.99–1.005	0.886
**VCAM-1**	**0.08** **±0.02**	**1.086**	**0.917**	**1.05–1.13**	**<0.001**
TnI	−0.16 ± 1.12	0.85	0.84	0.09–7.71	0.888

*CIH, Harrell concordance index; CI, confidence interval for HR; SE, standard error; ST2, soluble suppression of tumorigenicity 2; VCAM-1, vascular cells adhesion molecule; TnI, highly sensitive troponin I. The p < 0.05 is marked bold*.

**Figure 3:**
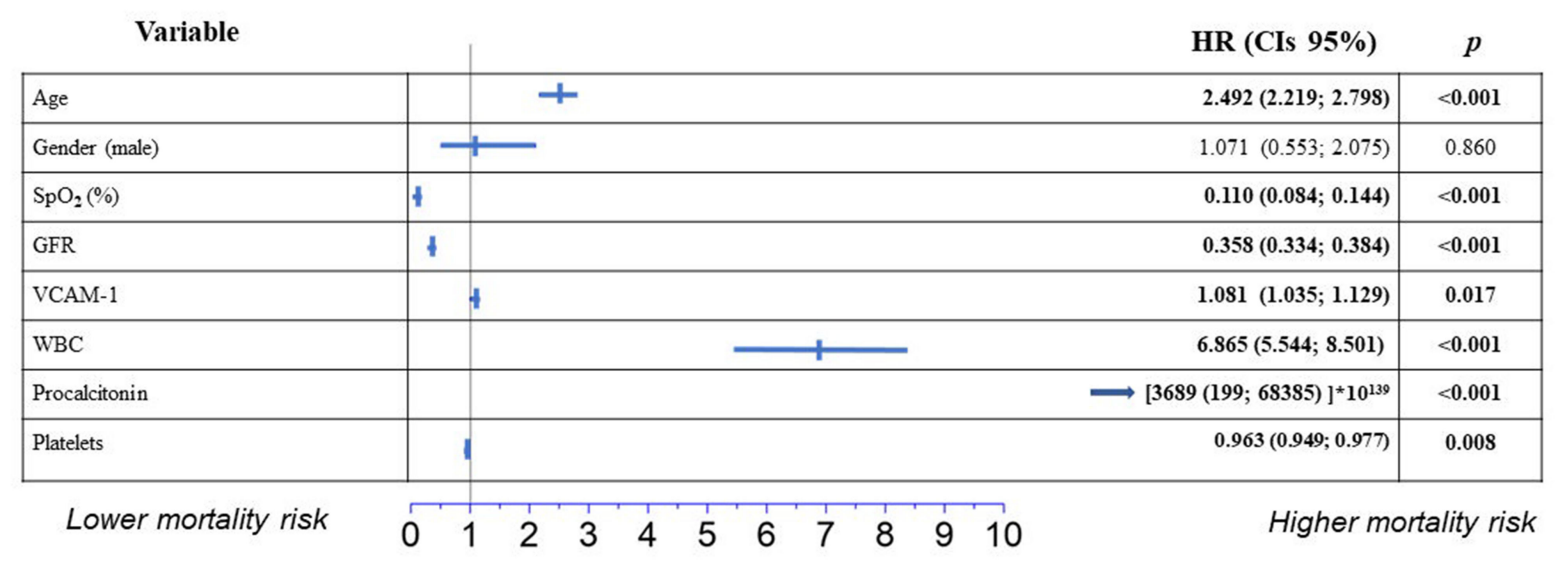
Independent predictors of hospital mortality from COVID-19 in multivariable survival regression (Bayesian Hierarchical Cox model) in a multi-marker model. Results are reported as hazard ratio (HR) and 95% confidence intervals (CIs).

The Gsslasso Cox Bayesian hierarchical model was based on identified risk factors for in-hospital mortality. It was constructed to assess their combined impact on survival in a multi-marker model. VCAM-1, age and gender as control variables were also added to the pool of risk factors to create the multi-marker model (Figure 3). By creating the preliminary multifactor model, risk factors arterial hypertension, coronary heart disease and chronic heart failure showed no relevant significance (*p* > 0.05) and were excluded from the model. Harrell's C-index (CIH) of the applied multi-marker model was 0.89, which indicates its satisfactory quality. On the other hand, while removing VCAM-1 from the model, it presented lower Harrell's C-index (CIH) of 0.814 with measure of explained risk of 0.83. Consequently, based on higher model quality metrics we continued by analyzing the model which included VCAM-1. With respect to the variables age, low SpO_2_, GFR and platelets, WBC and procalcitonin, VCAM-1 remained an indicator associated with fatal events, while the highest HR (3689 ^*^10^139^) was revealed for procalcitonin. In low SpO_2_, GFR and platelets variables HR was <1, which indicates that the lower range of the indicators increases in-hospital mortality (Figure 3).

### Post Discharge 1-Year Follow-Up Survival Analysis

Among the remaining 269 patients, FU analysis was performed from discharge to 366 days. Death was registered in 11 patients (4.1%; Table 4). The deceased patients were remarkably older: 73 (61; 82, *p* = 0.002) vs. 59 years (49; 66), and more often had arterial hypertension (*p* < 0.001) but had no difference in the clinical presentation as well as lower rates of diabetes mellitus (*p* < 0.001) and obstructive lung disease (*p* = 0.011). With respect to the investigated biomarkers, only ST2 was significantly higher in the deceased group (*p* = 0.024). Additionally, this group more often had cardiovascular (CV) and non-CV hospitalizations (*p* < 0.05, Table 4). The respective biomarkers were analyzed in a next step, with respect to the endpoint 1-year post-discharge mortality, where age was the control variable. Table 5 presents coefficients of Cox univariate regression proportional hazards for investigated mortality biomarkers. According to the univariant Cox regression models, the most accurate mortality risk predictors were ST2 (HR 1.004, *p* < 0.001) and TnI (HR 1.28, *p* = 0.011).

**Table 4:** Comparison of deceased and surviving patients at 1-year FU.

**Parameter**	**Survivors in 1-year FU,** **(Q1; Q3) or %**	**Deceased patients during 1-year FU (Q1; Q3) or %**	***P***
*n*, %	258 (95.9%)	11 (4.1%)	-
FU, days	**354.5 (342; 361)**	**50 (2; 146)**	**<0.001**
Gender, m/f	108/150 (41.9%/58.1%)	6/11 (55%/45%)	0.087
Age, years	**59 (49; 66)**	**73 (61; 82)**	**0.002**
BMI, kg/m^2^	27.9 (25.2; 32.3)	27.5 (26.9; 29.3)	0.854
***Clinical presentation at admission***			
SpO_2_, % Temperature at admission, °C SAP, mm Hg DAP, mm Hg HR, beats / min BR, breaths / min Lung tissue damage on CT, %	97 (95; 98) 36.5 (36.5; 37.5) 130 (140/120) 82 (72; 90) 91 (75.6; 102) 19 (18; 20) 36 (22; 52)	98 (96; 98.5) 36.7 (36.5; 36.8) 140 (156.5; 120) 88 (82.5; 91) 92.5 (79; 109) 20 (18; 20) 40 (22.5; 50)	0.313 0.315 0.240 0.217 0.772 0.645 0.662
***Relevant concomitant disease***			
AH, % (*n*) DM, % (*n*) CKD, % (*n*) CHD, % (*n*) CHF, % (*n*) History of MI, % (*n*) History of Stroke, % (*n*) Obstructive lung disease, % (*n*) AF, % (*n*)	**37.2 (96)** **77.5 (20)** 1.2 (3) 5.0 (13) 1.9 (5) 0 0 **8.1 (21)** 0	**80 (72.7)** **0** 0 9.1 (1) 0 0 0 **0** 0	**<** **0.001** **<** **0.001** 0.855 0.392 0.512 - - **0.011** -
***Laboratory parameters***			
Hb, dg/l WBC, *10^9^ Platelets, *10^9^ ESR, mm/sec CRP, mmol/l Procalcitonin, ng/ml Albumin, g/l CK, *n* (%) Urea, mmol/l Creatinine, mmol/l GFR, ml/min/m^2^ D-Dimer, ng/ml Sodium, mmol/l Potassium, mmol/l	129 (119; 137) 4.54 (3.6; 6.4) 226 (173; 277) 29 (18; 40) 41.8 (18.4; 77.9) 0.09 (0.05; 0.15) 40.3 (37.5; 42.4) 120 (72; 213) **5.33 (4.29; 6.17)** 85.6 (76.9; 96.3) 66.5 (57.6; 78.4) 505 (437; 573) 143 (141; 145) 4.2 (3.9; 4.4)	134 (123.5; 138) 5.26 (4.3; 8.3) 234 (165.5; 306) 40 (16.5; 47.5) 53.7 (25.6; 72.4) 0.226 (0.108; 0.263) 39.75 (38.1; 42.3) 190.5 (95.25; 294) **6.88 (6.2; 8.1)** 99.25 (83; 106.9) 57.7 (56.5; 67.2) 525 (0; 712.5) 142.5 (142; 144.75) 4.15 (3.93; 4.45)	0.448 0.154 0.819 0.442 0.658 0.032 0.793 0.201 **0.011** 0.098 0.217 0.459 0.865 0.805
***Serum cardiovascular biomarkers***			
ST2, ng/mL VCAM-1, pg/mL TnI, ng/mL	**51.38 (31.4; 76.8)** 13.7 (9.4; 16.7) 0.03 (0.01; 0.07)	**66.41 (57.0; 293.8)** 18.4 (10.3; 26.9) 0.03 (0.01; 0.165)	**0.024** 0.148 0.225
***FU events***			
All FU events (except death): Non-CV hospitalization, % (*n*) CV hospitalization Myocardial infarction Stroke Pulmonary embolism	**22.5 (58)** **14.7 (38)** **6.6 (17)** 0.3 (1) 0.3 (1) 0.3 (1)	**72.3 (8)** **36.4 (4)** **18.2 (2)** 0 18.2 (2) 0	**<0.001** **0.001** **0.023** >0.999 **<** **0.001** >0.999

*AH, arterial hypertension; BA, bronchial asthma; CK, creatine kinase; CHD, coronary heart disease; CHF, congestive heart failure; CKD, chronic kidney disease; CRP, C-reactive protein; CT computer tomography; CV, cardiovascular; DBP, diastolic blood pressure; DM, Diabetes Mellitus type 2; ESR, erythrocytes sedimentation rate; Hb, hemoglobin, HR, heart rate; SBP, systolic blood pressure; WBC, white blood count; CK,creatine kinase; VCAM-1-vascular cells adhesion molecule-1; ST2, suppression of tumorigenicity 2; TnI-highly sensitive Troponin I. The p <0.05 is marked bold*.

**Table 5:** Univariate Cox regression for biomarkers associated with post-hospital 1-year FU mortality.

**Biomarker**	**Coefficient ±SE**	**Hazard ratio**	**CIH**	**CI**	***P***
**ST2**	**0.004** **±0.001**	**1.004**	**0.818**	**1.002–1.006**	**<0.001**
VCAM-1	0.04 ± 0.03	1.042	0.830	0.98–1.11	0.169
**TnI**	**0.25** **±0.09**	**1.28**	**0.808**	**1.06–1.56**	**0.011**

*CIH, Harrell concordance index; CI, confidential interval for HR; SE, standard error; ST2, soluble suppression of tumorigenicity 2; VCAM-1, vascular cells adhesion molecule-1; TnI, highly sensitive troponin I. The p < 0.05 is marked bold*.

**Figure 4:**
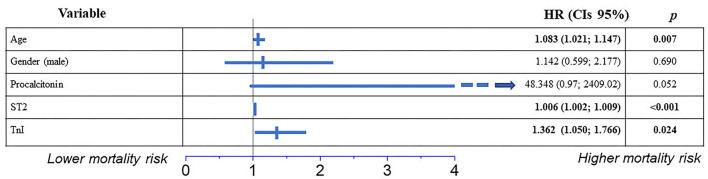
Independent predictors of post-hospital 1-year FU mortality in patients hospitalized with COVID-19, in a multivariate survival analysis using Cox's regression model. Results are reported as hazard ratios (HR) and 95% confidence intervals (CIs).

Using the Univariate Cox regression, we also analyzed the differences in survival rates of 1-year FU mortality in groups divided according to the presence / absence of risk factor based on the normal / out of range laboratory and clinical parameters. The following variables were shown to be associated with mortality with *p* < 0.1: procalcitonin, SpO2, urea, WBC, and arterial hypertension (Supplementary Table 4). The Gsslasso Cox Bayesian hierarchical model was constructed based on identified univariate risk factors to assess their combined impact on survival using a multi-marker model. The biomarkers ST2 and TnI as well as age and gender as a control variable were also added to the pool of risk factors to create a multi-marker model for survival. When using a preliminary multifactor model, the risk factors low SpO_2_, Urea, WBC, and arterial hypertension were not found to be significant (*p* > 0.1) and were thus excluded from the model. The Harrell's C-index (CIH) of the applied model was 0.856 with a measure of explained risk of 0.81, which indicates its satisfactory quality. While removing TnI and ST2 biomarkers from the model, Harrell's C-index (CIH) was 0.812 with the measure of explained risk of 0.730. Consequently, based on higher model quality metrics we continued by analyzing the model which included ST2 and TnI. Figure 4 presents the results of coefficients of the multivariate Bayesian Hierarchical Cox model for post-discharge all-cause mortality during 1-year FU. Age, TnI, and ST2 remained the indicators associated with post-discharge mortality during 1-year FU (Figure 4).

## Discussion

The emergence of COVID-19 has posed an unprecedented challenge to clinicians around the world. COVID-19 exhibits a wide clinical spectrum ranging from asymptomatic or mild respiratory tract symptoms to the development of acute respiratory distress syndrome and death. COVID-19 places a significant strain on healthcare systems, and diagnostic tools to guide decision-making to allocate potentially limited resources are still urgently needed. The disease was shown to be correlated to a high inflammatory burden, in part resulting in multi-organ damage and respiratory failure (25, 26). This systemic inflammatory response generates a “cytokine storm” (27), also affecting endothelial function, emphasized also by histological analyses (28, 29). Thus, a stratification of disease severity through vascular biomarkers seems plausible.

In COVID-19, several clinical parameters including laboratory parameters and radiographic findings were shown to help identify high-risk patients (1). Similarly, several clinical scores have been developed to predict the disease course of COVID-19 patients (30–32). The biomarkers most frequently included in these predictive models are typically indicators of cell damage (LDH, TnT/I) and inflammatory parameters (IL-6, ferritin, or lymphocytes count) (33, 34). Nevertheless, despite promising results, the suitability of biomarkers for the assessment of outcome in COVID-19 patients remains a matter of debate. Furthermore, to maximize diagnostic power, a multi-marker approach has been promoted and also shown to enhance the sensitivity and specificity of prognostic assessments (35).

With these previous studies in mind, we therefore aimed to assess the prognostic impact of the cardiac biomarker high-sensitive TnI, along with sST2 and VCAM-1, on in-hospital mortality as well as on 1-year post discharge survival after a hospitalization due to COVID-19 pneumonia. In-hospital mortality of COVID-19 patients was indeed associated with higher levels of VCAM-1 at hospital admission, while no correlation was evident for sST2 and high-sensitive TnI. Along with VCAM-1, typical risk factors such as old age, low SpO_2_, GFR and platelet count, high WBC, and high levels of procalcitonin were independent indicators of mortality. Previous studies suggested that VCAM-1, and intracellular cell adhesion molecule-1 (ICAM-1) might promote the interaction between leukocytes and endothelial cells, by serving as ligands for integrins, and thus may play an important role in COVID-19 pathogenesis (36, 37). Impaired endothelial activation can lead to high accumulation of leukocytes and enhanced transmission of intracellular signals, which can result in persistent systemic inflammation and viral-induced endothelial dysfunction. This may be an underlying, unifying mechanism responsible for the widespread systemic manifestations seen with SARSCoV-2 infection (38). On this regard, also term “Acute Vascular Distress Syndrome” was introduced, to account for the quite unique vascular pathophysiology in COVID-19 pneumonia (17, 18). A key feature reported is a low ventilation-to-perfusion ratio, leading to an increased pulmonary blood flow with intrapulmonary right to left shunt (18). This mechanism might also account for the incoherence of clinical and radiographic findings and the in part atypical clinical presentation of dyspnea in COVID-19 patients (18).

Similar to our findings, a recent meta-analysis comprising 349 critically ill and 337 non-critically ill patients described significantly higher rates of VCAM-1 levels in COVID-19 patients with a proposed cut-off point of 2523.7 ng/ml for critically ill and 1921.1 ng/ml for non-critically ill patients, respectively (21). Moreover, Bauer et al. (39) conducted a comparison of critically ill COVID-19 and non-COVID-19 patients requiring intensive care treatment, which again revealed significantly higher VCAM-1 levels in the COVID-19 group (39). Additionally, a direct correlation between VCAM-1 levels and viral RNA load in plasma was noted (40). Based on these and our findings, it can be speculated that vascular involvement is an important promoter of in-hospital mortality in COVID-19. This is also in line with cardio-embolic events as frequent complications especially in severe COVID-19 (41). Furthermore, the positive results of the Recovery trial along with the reduction of cardio-embolic events through use of dexamethasone therapy further emphasize the relevance of vascular complications in the acute setting of COVID-19 (42).

With regards, to high-sensitive TnI levels, we did not find an impact on prognosis. This finding stands in contrast to previous studies (43, 44). Still, the reasons for our contradictory results might be founded in our study design. On the one hand, the lack of a clear association between high-sensitive TnI and in-hospital mortality may partly be explained by the collection of blood samples at the 1st day of hospitalization. However, virally- induced cardiac injury usually requires 1–2 weeks to develop until clinical manifestations and resultant high-sensitive TnI increases occur (5, 45). This is further emphasized by a study of Zhou et al. (5), in which serum high-sensitive TnI median concentrations increased from 57.6 to 290.6 pg/mL during the period between day 16 and day 22 after the onset of COVID-19 infection in non-survivors (5). Similar effects might be responsible for the findings of sST2. On the other hand, high-sensitive TnI levels showed a strong trend toward an increase in non-survivors, with a *p*-value of 0.050. Accordingly, the lack of prognostic impact might also be attributed to the small sample size of our study, which is a limitation of our study design.

Accordingly, while VCAM-1 had the best prognostic power in the assessment of in-hospital mortality, levels of sST2 were of significant prognostic value with regard to long-term prognosis. This correlates to the results of recent studies which report a significant increase in cardiovascular disease burden over 1-year follow up in COVID-19 survivors (46). Accordingly, the numbers of CV as well as non-CV hospitalizations and stroke were significantly higher in the deceased group (*p* < 0.05). Similar to our findings, a prognostic benefit of sST2 with respect to disease severity and mortality was also shown in a former study of 100 hospitalized COVID-19 patients (47). As soluble ST2 represents a marker of inflammation and cardiac stress, the reasons for this finding may be diverse (9). For one, higher sST2 levels might be triggered by a higher inflammatory burden (16), potentially resulting in ongoing inflammation, or even virus persistence and long-COVID-19 syndrome. Similarly, higher levels of sST2 might be promoted by diverse comorbidities. sST2 was shown to be an effective prognostic tool in long-term risk stratification in patients with heart failure, myocardial infarction, and stable coronary heart disease (48). Thus, by incorporating different pathophysiological processes, higher levels of sST2 might also point toward a more ill patient collective. The higher rates of CV and non-cardiovascular hospitalizations and stroke in the deceased group during FU (*p* < 0.001) further supports this suggestion. Moreover, the association of high-sensitive TnI with FU mortality matches previous studies, reporting a correlation of higher TnI/T concentrations with subsequent cardiovascular endpoints including heart failure decompensation, myocardial infarction, and viral myocarditis (49, 50).

With regards to the observed differences regarding short- and long-term prognosis, also the pathophysiological mechanisms have to be considered. VCAM-1 and ICAM-1 as well as sST2 represent circulating biomarkers (43, 44). However, circulating levels might still display differences with regards to their cellular, membrane bound forms. Given the fact, that VCAM-1 acts as a cell adhesion molecule in the context of inflammatory processes, a fast effect of an increase in VCAM-1 can be assumed (51, 52). Of note, changes in levels of VCAM-1 were reported in a comparably short timespan of days to hours (51, 52). Thus, VCAM-1 might represent a promising parameter reflecting short term effects in COVID-19, while long-term prognostic impact is limited. On the other hand, expression of sST2 is influenced by numerous comorbidities, thus reflecting an overall health status, not necessarily limited to ongoing inflammatory processes (53–55). While this might limit its impact on short-time prognosis, the incorporation of different pathophysiologic processes makes it a suitable marker for long term prognosis such as in the context of COVID-19 (53–55). From a cardiovascular aspect, sST2 further represents a marker of cardiac fibrosis and was shown to be elevated in heart failure (55). While cardiac injury was reported in the context of COVID-19, cardiac remodeling and cardiac fibrosis itself represent an ongoing process over months and years. Accordingly, worse outcomes due to cardiac fibrosis and remodeling might primarily induce undesirable long-term effects after a COVID-19 infection.

In conclusion, our study demonstrates the potential of novel cardiovascular biomarkers in the context of COVID-19. Based on the pathophysiological processes involved, VCAM-1 represents a promising prognostic marker for the assessment of in-hospital mortality in COVID-19. On the other hand, sST2 as well as high-sensitive Tn I provided prognostic value in the long-term follow-up.

### Limitations

The greatest limitation of our study is the single-center design along with a comparably small sample size. This might limit the significance of our results. Thus, the findings of our study have to be considered as primarily hypothesis generating. Furthermore, the rapid evolution of COVID-19 management during the time of biomarker collection (June to August 2020) should be taken into consideration. Cardiac imaging assessments were not routinely performed in our study. Of note, advanced cardiac imaging including echocardiography, would have provided important information about potential correlations between cardiac functional impairments and the investigated biomarkers. Since only hospitalized patients were included, the results cannot be transferred to milder COVID-19 disease. Moreover, biomarkers were measured only at admission, and no FU values were assessed. Hence, no conclusions with regards to the role of tested biomarkers as potential tools for disease and therapy monitoring can be drawn. Accordingly, our data are only representative for their prognostic ability at baseline. Therefore, despite promising results, routine application of the proposed multi-marker approaches may be limited.

## Data Availability Statement

The raw data supporting the conclusions of this article will be made available by the authors, without undue reservation.

## Ethics Statement

The studies involving human participants were reviewed and approved by Ethic committee of the Bashkir State Medical University (N5, 2020). The patients/participants provided their written informed consent to participate in this study.

## Author Contributions

LM, PJ, DG, PD, RG, IL, AT, RZ, IK, BC, BV, VP, KK, UH, ML, LF, RP, and NZ meet the criteria for authorship and contributorship as defined by the ICMJE. All authors contributed to the article and approved the submitted version.

## Funding

This study was supported by grant of Russian Science Foundation No 22-18-20123.

## Conflict of Interest

The authors declare that the research was conducted in the absence of any commercial or financial relationships that could be construed as a potential conflict of interest.

## Publisher's Note

All claims expressed in this article are solely those of the authors and do not necessarily represent those of their affiliated organizations, or those of the publisher, the editors and the reviewers. Any product that may be evaluated in this article, or claim that may be made by its manufacturer, is not guaranteed or endorsed by the publisher.
