# Supplementary material for: Cardiovascular Biomarkers for Prediction of in-hospital and 1-Year Post-discharge Mortality in Patients With COVID-19 Pneumonia
Source: Front Med (Lausanne). 2022 Jun 28;9:906665. doi: 10.3389/fmed.2022.906665 (PMC9273888; doi:10.3389/fmed.2022.906665)
Supplement: Supplementary file 1 [file Data_Sheet_1.docx]

**Supplemental Tables**

**Supplemental Table 1**. COVID-19 relevant in-hospital therapy

| Parameter | All |
| --- | --- |
| N | 280 |
| Hydroxychloroquine, % (n) | 56.8 (159) |
| Lopinavir and Ritonavir, % (n) | 36.1 (101) |
| Tozilisumab, % (n) | 8.9 (25) |
| Glucocorticosteroids, % (n) | 100 (280) |
| Therapeutic low-molecular-weight heparin, % (n) | 96.8 (271) |

**Supplemental Table 2**. Relevant post-discharge therapy

| Parameter | All |
| --- | --- |
| N | 268 |
| Beta-blockers, % (n) | 8.9 (25) |
| Diuretics, % (n) | 4.3 (12) |
| ACE inhibitor / ARA, % (n) | 25.4 (71) |
| Warfarin, % (n) | 0.4 (1) |
| Aldosterone antagonists, % (n) | 2.1 (6) |
| Statins, % (n) | 8.6 (24) |

ACE-angiotensin converting enzyme, ARA-angiotensin receptor antagonist

**Supplemental table 3.** Univariate Cox regression for in hospital mortality risk factors in COVID-19 patients with p<0.1

| **Parameter** | **Coefficient** | **SE** | ***P*** |
| --- | --- | --- | --- |
| BMI | 0.048 | 0.052 | 0.36 |
| **SpO_2_** | **0.118** | **0.070** | **0.092** |
| T, °C | 0.022 | 0.479 | 0.963 |
| SBP | 0.025 | 0.022 | 0.257 |
| DBP | 0.001 | 0.042 | 0.978 |
| HR | 0.009 | 0.027 | 0.738 |
| BR | 0.031 | 0.298 | 0.916 |
| Hb | 0.031 | 0.028 | 0.272 |
| WBC | 0.072 | 0.071 | 0.312 |
| Platelets | 0.009 | 0.007 | 0.203 |
| ESR | 0.018 | 0.030 | 0.542 |
| CRP | 0.010 | 0.016 | 0.537 |
| **Procalcitonin** | **2.465** | **0.737** | **<0.001** |
| Albumin | 0.008 | 0.093 | 0.932 |
| CK | 0.001 | 0.001 | 0.904 |
| **GFR** | **-0.089** | **0.029** | **0.002** |
| Sodium | 0.1224 | 0.107 | 0.254 |
| Potassium | -0.025 | 0.164 | 0.877 |
| **AH** | **2.047** | **1.095** | **0.062** |
| DM | 0.913 | 1.096 | 0.404 |
| **CKD** | **2.638** | **1.096** | **0.016** |
| **CHD** | **3.480** | **0.867** | **<0.001** |
| **CHF** | **2.210** | **1.096** | **0.044** |

AH–arterial hypertension, BA–bronchial asthma, CK–creatine kinase, CHD–coronary heart disease, CHF-congestive heart failure, CKD–chronic kidney disease, CRP- C-reactive protein, CT computer tomography, DBP–diastolic blood pressure, DM–Diabetes Mellitus, ESR–erythrocytes sedimentation rate, Hb–hemoglobin, HR–heart rate, MI–myocardial infarction, MV-mechanical ventilation, NIV-noninvasive ventilation, SBP–systolic blood pressure, CK—creatine kinase, ST2 - suppression of tumorigenicity 2, TnI – highly sensitive Troponin I, VCAM-1— vascular cells adhesion molecule-1, WBC–white blood count.

**Supplemental table 4.** Univariate Cox regression for post-discharge 1-year follow-up mortality in COVID-19 patients with p<0.1

| **Parameter** | **Coefficient** | **SE** | ***P*** |
| --- | --- | --- | --- |
| BMI | -0.013 | 0.058 | 0.829 |
| **SpO_2_** | **-0.191** | **0.080** | **0.017** |
| T, °C | -0.415 | 0.364 | 0.254 |
| SBP | 0.025 | 0.017 | 0.137 |
| DBP | 0.037 | 0.031 | 0.236 |
| HR | 0.006 | 0.021 | 0.77 |
| BR | 0.041 | 0.218 | 0.853 |
| Hb | 0.008 | 0.020 | 0.68 |
| **WBC** | **0.089** | **0.050** | **0.074** |
| Platelets | 0.002 | 0.004 | 0.61 |
| ESR | 0.018 | 0.873 | 0.383 |
| CRP | 0.005 | 0.012 | 0.686 |
| **Procalcitonin** | **2.952** | **1.739** | **0.089** |
| Albumin | -0.018 | 0.070 | 0.793 |
| CK | -0.00002 | 0.0003 | 0.958 |
| **Urea** | **0.352** | **0.148** | **0.018** |
| Creatinine | 0.015 | 0.012 | 0.226 |
| GFR | -0.022 | 0.021 | 0.288 |
| Sodium | 0.037 | 0.097 | 0.702 |
| Potassium | -0.055 | 0.210 | 0.792 |
| **AH** | **1.453** | **0.677** | **0.032** |
| DM | -17.13 | 547.1 | 0.998 |
| CKD | -16.02 | 828.1 | 0.998 |
| CHD | 0.670 | 1.049 | 0.523 |
| CHF | 16.03 | 665.0 | 0.998 |

AH–arterial hypertension, BA–bronchial asthma, CK–creatine kinase, CHD–coronary heart disease, CHF-congestive heart failure, CKD–chronic kidney disease, CRP- C-reactive protein, CT computer tomography, DBP–diastolic blood pressure, DM–Diabetes Mellitus, ESR–erythrocytes sedimentation rate, Hb–hemoglobin, HR–heart rate, MI–myocardial infarction, MV-mechanical ventilation, NIV-noninvasive ventilation, SBP–systolic blood pressure, CK—creatine kinase, ST2 - suppression of tumorigenicity 2, TnI – highly sensitive Troponin I, VCAM-1— vascular cells adhesion molecule-1, WBC–white blood count.

**Supplemental table 5.** Available echocardiographic data during in-hospital period and during 1-year follow-up of the investigated COVID-19 patients.

| **Parameter** | **Parameters, median (Q1;Q3)** |
| --- | --- |
| **During in-hospital period (n=12)** |  |
| Time from hospitalization, days | 3 (2,3; 3,6) |
| LVEF, % | 65.0 (57.0; 66,25) |
| LVEDD, mm | 4.8 (4.5; 5.35) |
| LVESD, mm | 3.9 (3.6; 4.2) |
| **During 1-year follow-up (n=19)** |  |
| Time from hospitalization, days | 127 (102; 167) |
| LVEF, % | 66 (62.0; 70) |
| LVEDD, mm | 4.5 (4.18; 4.8) |
| LVESD, mm | 2.9 (2.6; 3.3) |

Echocardiography was only performed based on physician´s decision (if regarded clinically necessary by the physician in charge). Therefore, echocardiography data was only available in 12 patients during the in-hospital period and in 19 patients during 1-year follow-up.

LVEDD – left ventricular end-diastolic diameter, LVEF – left ventriclular ejection fraction, LVESD – left ventricular end-systolic diameter
